# Bloody Phenomenon: A Rare Case of Hemobilia From Cystic Artery Hemorrhage Following Percutaneous Cholecystostomy

**DOI:** 10.7759/cureus.99625

**Published:** 2025-12-19

**Authors:** Sparkle R Tonge, Abdul H Khan, Michael R Zemaitis

**Affiliations:** 1 General Surgery, American University of Antigua, Osbourn, ATG; 2 General Surgery, Richmond University Medical Center, Staten Island, USA

**Keywords:** biliary bleeding, biliary stent migration, covered stent angioplasty, cystic artery hemorrhage, emphysematous cholecystitis, endoscopic retrograde cholangio-pancreatography, hepatobiliary intervention, interventional radiology, percutaneous cholecystostomy, post-surgical hemobilia

## Abstract

Hemobilia, defined as bleeding into the biliary tract, is a rare but potentially life-threatening condition. It most commonly results from trauma, malignancy, or iatrogenic interventions; however, its clinical presentation is often atypical and frequently lacks the classic Quincke's triad, making the diagnosis challenging and potentially leading to delays in management and treatment. With the increasing use of percutaneous cholecystostomy in high-risk patients with cholecystitis, there is a growing recognition of vascular complications such as cystic artery hemorrhage.

We describe the case of a 72-year-old male with a complex cardiovascular and metabolic history who presented with sanguineous drainage from a percutaneous cholecystostomy tube, three weeks after undergoing endoscopic retrograde cholangiopancreatography (ERCP) with common bile duct (CBD) stone removal and biliary stent placement. He had developed a perihepatic collection requiring cholecystostomy during his prior hospitalization. On re-presentation, he exhibited mild abdominal tenderness, leukocytosis, anemia, and hypoglycemia. CT angiography revealed active extravasation from a branch of the cystic artery and a displaced CBD stent. The patient underwent a successful angiogram with placement of a covered stent in the right replaced hepatic artery. He was managed in the intensive care unit with broad-spectrum antibiotics, bowel rest, glycemic control, and supportive care. Following clinical improvement, he was discharged to a rehabilitation center with plans for elective cholecystectomy and stent reassessment.

This case discusses the importance of recognizing hemobilia as a rare but serious and possible complication of percutaneous biliary drainage. Cystic artery hemorrhage may arise from mechanical trauma, inflammatory vascular erosion, stent migration, anatomical variations, or infection. Timely imaging and intervention, particularly via angiography, are essential for hemodynamic stabilization. Definitive management with cholecystectomy is key to preventing recurrence and further complications that may arise.

Clinicians should consider and maintain a high index of suspicion for hemobilia in patients presenting with bloody cholecystostomy output, even in the absence of classic signs such as gastrointestinal bleeding. Prompt imaging, multidisciplinary management, and definitive surgical management such as cholecystectomy are essential for preventing recurrence and further complications, thereby improving overall patient outcomes.

## Introduction

Hemobilia, or hemorrhage from the biliary tract, is an uncommon but potentially life-threatening complication most often associated with trauma, malignancy, vascular malformations, or hepatobiliary procedures. With the increasing use of interventional techniques such as endoscopic retrograde cholangiopancreatography (ERCP), liver biopsy, or percutaneous drainage procedures, iatrogenic vascular injuries, particularly involving the cystic artery or its branches, have emerged as important causes of upper gastrointestinal bleeding or biliary tract hemorrhage, although the overall incidence remains low [1]. The underlying mechanism typically involves arterio-biliary fistula formation or vessel erosion secondary to inflammation, instrumentation, or direct vascular injury. Clinically, patients may present with Quincke's triad, which involves right upper quadrant pain, jaundice, and gastrointestinal bleeding. However, this classic presentation occurs in fewer than 40% of cases [1]. Diagnosis may be challenging and often requires a combination of laboratory studies, cross-sectional imaging, angiography, and endoscopy. Management involves stabilization, identifying the source of bleeding in addition to control of hemorrhage, most commonly via angiographic embolization, while surgery is reserved for complex anatomy or refractory cases. Although hemobilia has been increasingly recognized as a complication of hepatobiliary procedures, bleeding originating specifically from the cystic artery remains exceedingly rare. In most reported cases, hemobilia has been identified following laparoscopic cholecystectomy, as a result of blunt or penetrating hepatic injuries, or secondary to endoscopic manipulation through ERCP [1-7]. In contrast, this case highlights a rare and underreported scenario of cystic artery hemorrhage resulting in hemobilia following placement of a percutaneous cholecystostomy tube, highlighting an uncommon source of biliary tract bleeding in the absence of recent surgical intervention.

## Case presentation

A 72-year-old male with a complex medical history, including congestive heart failure, chronic obstructive pulmonary disease (COPD), prior myocardial infarction complicated by cardiac arrest, coronary artery bypass grafting (CABG), type 2 diabetes mellitus, and hypertension, for which he was on medications including aspirin, atorvastatin, and furosemide, presented to the emergency department with complaints of bloody output from a cholecystostomy tube and mild abdominal discomfort in the right upper quadrant. The patient denied fever, chills, and abdominal pain; however, he reported one episode of non-bilious vomiting.

The patient was recently hospitalized for cholecystitis and choledocholithiasis. He underwent endoscopic retrograde cholangiography (ERCP), sphincterotomy, common bile duct (CBD) stone extraction, and stent placement. Post-procedure, his course was complicated by respiratory failure requiring intubation. Furthermore, a CT scan of the abdomen revealed a distended gallbladder with a perihepatic collection, suggestive of gallbladder perforation. This finding led to the placement of two tubes, one within the gallbladder and another within the perihepatic collection. This subsequently resulted in percutaneous cholecystostomy tube placement using an 8 French pigtail catheter. In addition, he underwent exchange and upsizing of the cholecystostomy drain to a ten French catheter during the same admission due to inadequate drainage and severance of the tube related to the location of the collection. He was subsequently discharged. The patient returned three weeks later with new-onset bloody output from his biliary drain.

On initial evaluation, the patient was afebrile with a temperature of 97.1°F, heart rate of 100 beats per minute, blood pressure of 108/49 mmHg with a mean arterial pressure of 68 mmHg, respiratory rate of 20 breaths per minute, and oxygen saturation of 97% on three liters of oxygen via nasal cannula. He appeared in no acute distress and was alert and oriented. Cardiopulmonary examination was unremarkable, with no murmurs or signs of fluid overload. Abdominal examination revealed mild tenderness in the right upper quadrant at the site of the cholecystostomy tube, without guarding, rebound, or organomegaly. The remainder of the physical examination was within normal limits.

**Table 1:** Laboratory values procured in the emergency department

Investigation	Patient Laboratory Values	Reference Ranges
WBC	19.1 k/μL	4.0–11.2 k/μL
Hemoglobin	9.7 g/dL	13.7–17.5 g/dL
Hematocrit	32.0%	40.0–51.0%
Mean corpuscular value	76.0 fL	79–98 fL
Neutrophils (%)	85.1%	30–71%
Absolute neutrophils	16.28 k/μL	1.8–6.5 k/μL
Prothrombin time	15.1 seconds	12–14.8 seconds
International normalized ratio	1.20	0.9–1.12
Activated partial thromboplastin time	37.0 seconds	22.8–36.5 seconds
Serum glucose	42 mg/dL	74–106 mg/dL
Total bilirubin	1.9 mg/dL	0.2–1.0 mg/dL
Aspartate aminotransferase	210 U/L	<34 U/L
Alanine aminotransferase	156 U/L	10–49 U/L

**Figure 1:**
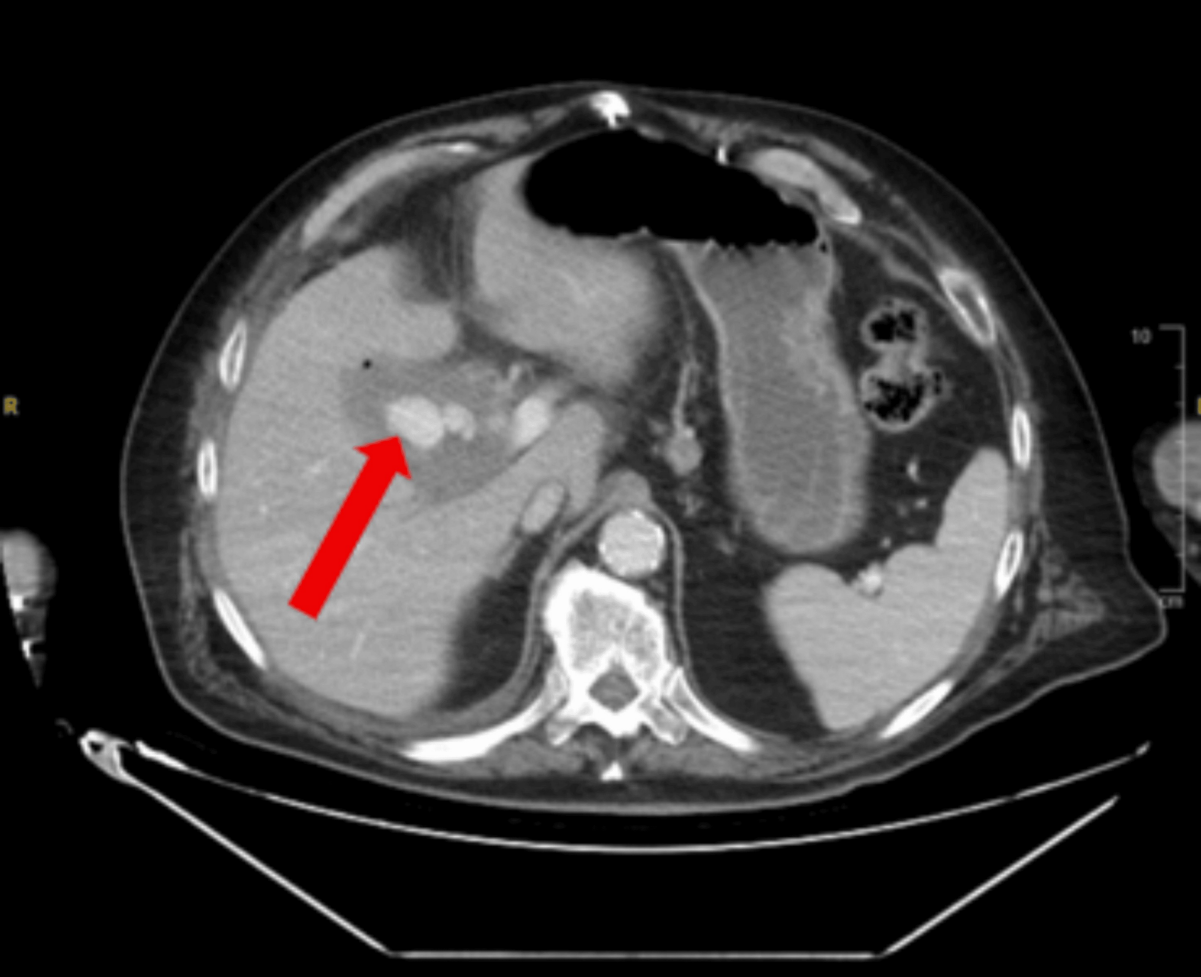
Axial CT angiogram of the abdomen and pelvis demonstrating active contrast extravasation (red arrow) from the branch of the right replaced hepatic artery into the gallbladder, showing multiple round areas of increased densities consistent with hemmorhage

**Figure 2:**
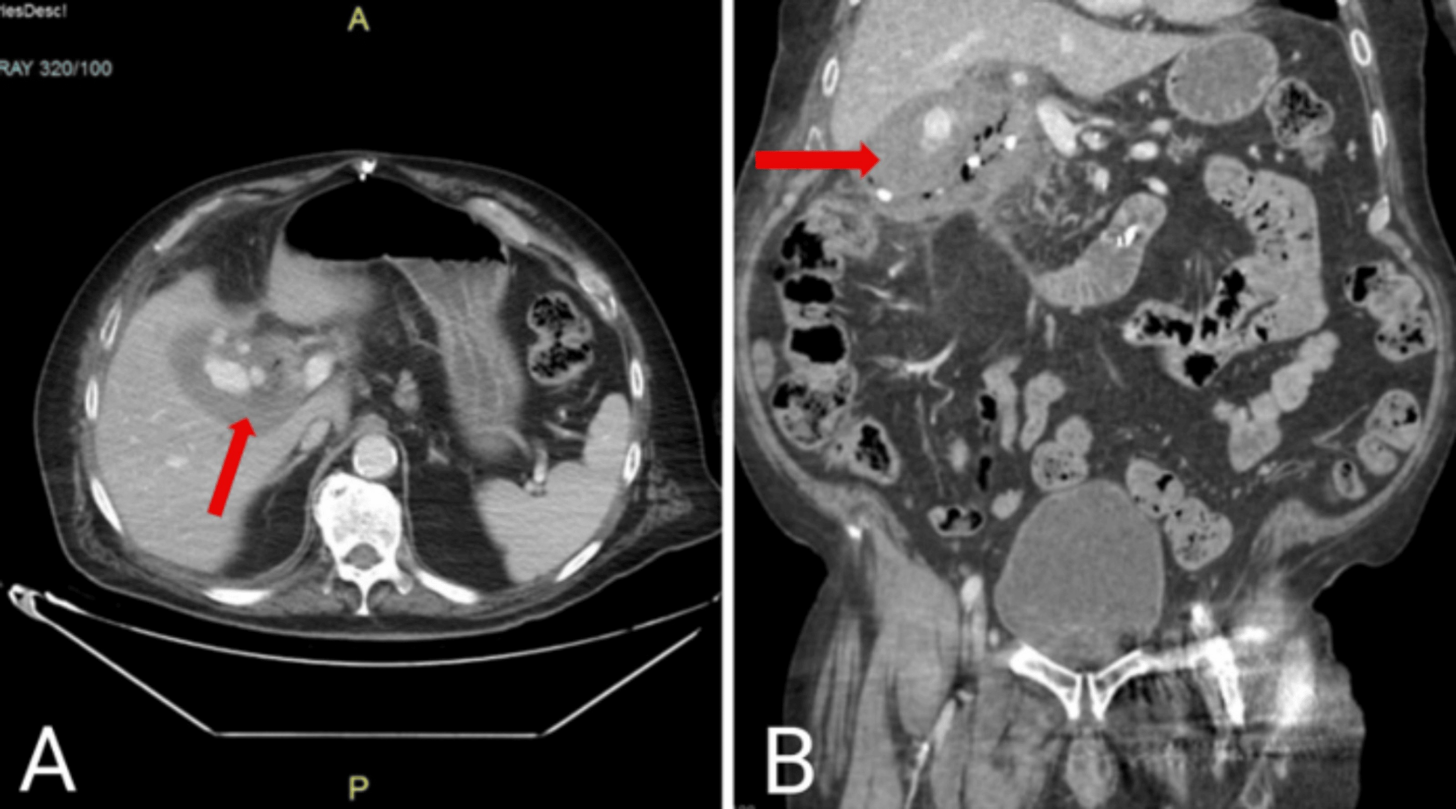
CT angiogram with IV contrast showing diffusely emphysematous gallbladder with edematous wall and patchy enhancement (red arrows) pattern consistent with cholecystitis (A) Axial. (B) Coronal.

**Figure 3:**
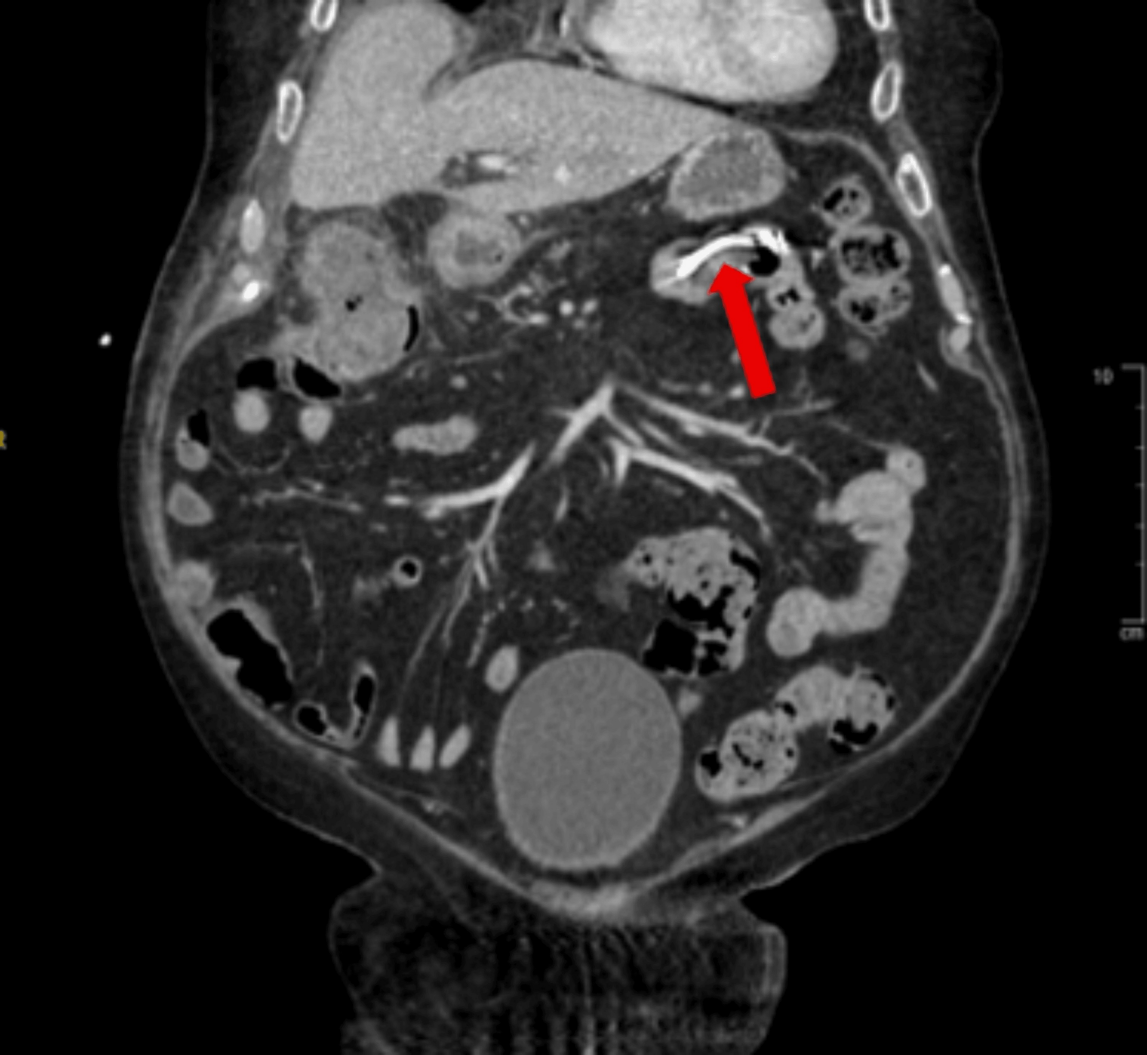
Coronal CT showing common bile duct stent (red arrow) visualized in the distal duodenum and proximal jejunum, indicative of migration.

A computed tomography angiogram of the abdomen and pelvis with intravenous contrast was obtained. The findings revealed active contrast extravasation from a branch of the replaced right hepatic artery into the gallbladder, which was concerning for arterial hemorrhage (Figure 1). The gallbladder appeared diffusely emphysematous, with wall thickening and patchy enhancement, concerning for cholecystitis (Figure 2). The previously placed CBD stent was visualized in the distal duodenum and proximal jejunum, indicative of stent migration (Figure 3). 

**Figure 4:**
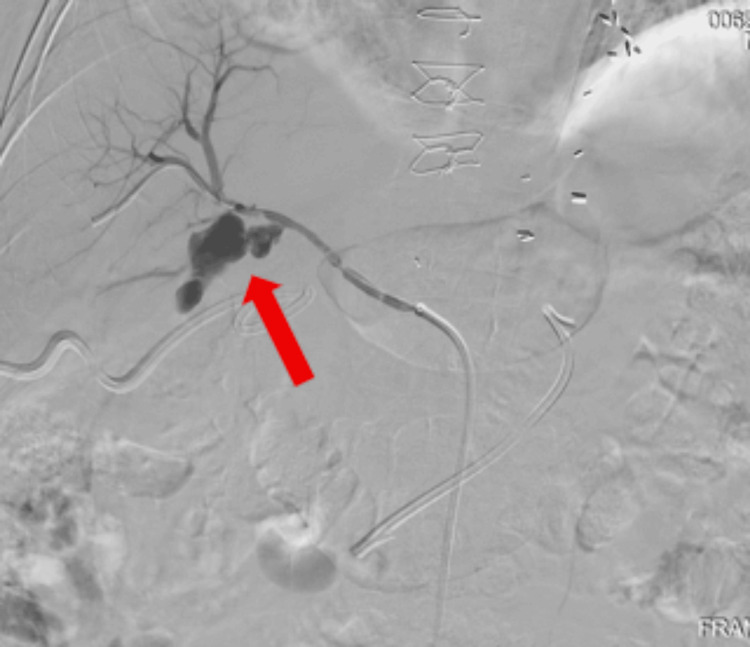
Angiogram demonstrating contrast extravasation from the cystic artery (red arrow)

Angioembolization was attempted; however, the cystic duct could not be cannulated during the procedure. Therefore, an emergent angiogram was performed, which confirmed active hemorrhage in the cystic artery (Figure 4). Hemostasis was achieved via placement of a 6 mm x 7.5 cm covered stent with preserved perfusion to the hepatic branches. A second 8 French cholecystostomy tube was placed for additional decompression. Evaluation via fluoroscopy revealed a patent bile duct without fistulous communication to the adjacent bowel.

Given his advanced age, cardiovascular disease, and ongoing sepsis, the team opted for conservative management, as he was considered a poor surgical candidate during that presentation. Treatment included broad-spectrum antibiotics, including linezolid and meropenem. Supportive care was also provided, including intravenous fluids, bowel rest, glucose repletion, and pain management with analgesics. Serial monitoring showed progressive resolution of leukocytosis, with WBC decreasing from 19.1 to 11 k/uL, and the patient remained hemodynamically stable.

Once clinically improved, the patient was transferred to the medical floor and subsequently discharged to a rehabilitation center. At discharge, the patient was tolerating oral intake, off supplemental oxygen, and reported significant improvement in abdominal pain. Follow-up with an outpatient hepatobiliary surgeon for consideration of elective cholecystectomy was arranged, along with gastroenterology follow-up for potential repeat ERCP to retrieve or replace the migrated CBD stent.

## Discussion

Hemorrhage from the cystic artery, although rare, can lead to detrimental complications that often arise in the setting of hepatobiliary interventions, as seen in the presented case, in which the patient developed emphysematous cholecystitis after undergoing ERCP with sphincterotomy, common bile duct stone extraction, and percutaneous cholecystostomy [1,2]. The subsequent bleeding was confirmed to originate from a branch of the cystic artery. This required an emergent angiogram in addition to supportive intensive care, demonstrating the complexity and severity of such a vascular event.

The literature reveals a limited number of documented cases involving delayed hemobilia following percutaneous cholecystostomy, particularly in patients managed nonoperatively. Additionally, with the increasing use of the percutaneous approach in patients with higher operative risk, this case offers a rare perspective on vascular complications associated with a procedure considered less invasive than laparoscopic cholecystectomy [7]. Most published reports have focused on hemobilia associated with laparoscopic cholecystectomy or cholecystostomy. In the present case, involvement of a replaced right hepatic artery, migrated biliary stent, emphysematous cholecystitis, and delayed onset of bleeding further demonstrated the complexity of the presentation. Furthermore, this case illustrated a nonclassical presentation in which bleeding was identified through cholecystostomy output rather than hematemesis or melena. It also highlights successful hemorrhage control using covered stent technology when standard embolization failed. Given this atypical manifestation, a high index of clinical suspicion is required in similar scenarios, underscoring the importance of considering hemobilia even in the absence of classic gastrointestinal symptoms. We report this case to raise awareness of this less common complication in nonsurgical patients.

Etiologies of cystic artery bleeding

The cystic artery is a branch of the right hepatic artery in approximately 79-89% of individuals. Due to its close anatomical relation to the gallbladder and biliary tree, it is vulnerable to iatrogenic injury. Spontaneous cystic artery bleeding is uncommon, with less than 100 cases and a handful reported in nontraumatic and traumatic conditions, respectively [8]. However, there are several factors that may account for such a presentation in this patient.

Iatrogenic Injury Post-ERCP

The occurrence of vascular injury associated with ERCP is infrequent, with the leading cause of iatrogenic injury being laparoscopic cholecystectomy [8]. However, it has been increasingly recognized with the use of expanded endoscopic interventions. Instances such as thermal injury during sphincterotomy, perforation from guidewire use, or increased intraluminal pressure from contrast injection can lead to disruption of the integrity of the artery, which may potentially trigger hemorrhage into the biliary tract (hemobilia) and adjacent tissues [1,8-9].

Anatomical Variation

Variations in anatomy regarding the cystic artery are common and can increase the rate of complications associated with hepatobiliary interventions. The artery may present with variable origins, lengths, or even multiple branches. It most often arises from the right hepatic artery and passes through Calot’s triangle. However, it may also originate from the gastroduodenal, left hepatic, or superior mesenteric arteries. In this patient, a replaced right hepatic artery was identified, thus displacing the cystic artery, which may have increased susceptibility to vascular injury and contributed to the atypical bleeding pattern [8].

Post-cholecystostomy Trauma

Percutaneous cholecystostomy is a therapeutic option for high-risk surgical patients presenting with acute cholecystitis. It involves transhepatic or transperitoneal access. Although it is minimally invasive in nature, complications can arise, such as friction from the catheter, inadvertent arterial puncture, or subsequent upsizing of the drain, which may compromise nearby vessels, such as the cystic artery or branches of the hepatic artery, and may be an etiological factor in this case [10].

Infectious and Inflammatory Vascular Erosion

Imaging findings in this case suggested emphysematous cholecystitis (Figures 3, 4). The occurrence of emphysematous cholecystitis is about 1% of all cases of acute cholecystitis [11]. Individuals susceptible to this phenomenon include persons with diabetes mellitus and those who are immunocompromised, such as this patient. This may lead to transmural necrosis and cystic artery wall erosion. Invasion by bacteria, notably *Clostridium*, *E. coli*, and *Klebsiella*, can promote enzymatic degradation of the vessel walls, which can lead to the formation of a pseudoaneurysm or frank rupture [8,11].

Dislodged CBD Stent

Mechanical trauma to the mucosa and adjacent vascular structures has been implicated in the migration of biliary stents. Most commonly, this is known to cause mucosal ulcerations, cholangitis, and pancreatitis [12]. However, erosion into the wall of the cystic artery is plausible, especially in a previously inflamed and edematous hepatobiliary tract, as observed in this patient.

Antiplatelet Therapy

Bleeding may have been potentiated due to the patient’s chronic use of low-dose aspirin. Aspirin may exacerbate any disruption of the microvasculature, although it is necessary for secondary cardiovascular prevention. This may contribute to persistent hemorrhage, particularly in the setting of sepsis or coagulopathy [13].

Clinical manifestations and diagnostic workup

The presentation of hemobilia is known by Quincke’s triad, which encompasses right upper quadrant pain, gastrointestinal bleeding, and jaundice, though all three are not commonly seen, with an occurrence of 22%-35% in patients with hemobilia [1]. In this case, the bleeding was identified via cholecystostomy output rather than hematemesis or melena. This demonstrates the need for high clinical suspicion in atypical presentations. Furthermore, laboratory studies revealed anemia, mild coagulopathy, and leukocytosis, all of which can make the diagnosis uncertain if not confirmed by imaging.

CT angiography is the hallmark of diagnosis, as it offers both localization of the anatomical structures and assessment of contrast extravasation in hemodynamically stable patients in 99% of cases [8,12]. In our case, there was visualization of active bleeding from the region of the cystic artery, thus prompting interventional management. The decision by the team to proceed directly to CT angiography, rather than initial endoscopy, proved optimal due to the external route of drainage and the need for mapping of the vasculature prior to intervention. Other alternative approaches include upper endoscopy, which may be considered when hemobilia presents with hematemesis or melena. However, endoscopy cannot localize or assess vascular anatomy [1].

Management strategies

Angiogram With Stent Placement

The gold standard for the management of visceral arterial hemorrhage in hemodynamically stable patients is transcatheter arterial embolization [8]. Selective embolization of the cystic artery was attempted and could not be achieved due to technical challenges, likely related to the patient’s variant anatomy with a replaced right hepatic artery and acute inflammatory changes surrounding the vessel. However, alternate procedures can be utilized and remain efficacious. In this patient, bleeding was successfully arrested via angiography using a 6 mm × 7.5 cm covered stent while preserving hepatic perfusion. It was used as both a diagnostic and therapeutic intervention. An advantage of using covered stents is that they maintain arterial patency of the replaced right hepatic artery while excluding the bleeding source. Additionally, the use of a covered stent, instead of traditional embolization techniques for hemostasis, may provide an important procedural insight in the literature, as embolization is the preferred method to achieve devascularization for hemostasis [8,14,15].

The most significant management decision in this case was the choice to pursue conservative management rather than urgent or semiurgent surgical intervention during the initial admission. The patient’s significant cardiovascular comorbidities, which included prior CABG and myocardial infarction, in addition to advanced age and ongoing sepsis, rendered him an extremely poor surgical candidate with increased perioperative morbidity and mortality risk for emergency cholecystectomy. This ultimately led to the attempted embolization procedure and the subsequent placement of the covered stent.

Supportive and Adjunctive Care

Broad-spectrum antibiotics were essential given the suspected cholecystitis with emphysematous changes and perihepatic collection. Linezolid and meropenem provided antimicrobial coverage for gram-negative enteric organisms such as E. coli and Klebsiella, anaerobes including Clostridium species, and methicillin-resistant Staphylococcus aureus [11]. Blood transfusion and IV fluids were guided by clinical status and hemoglobin trends. Addressing the severe hypoglycemia (42 mg/dL) was a critical step in management, followed by tight glycemic control throughout the ICU stay given the patient’s history of diabetes. Perioperative hyperglycemia, with glucose levels >180 mg/dL, is associated with increased surgical site infections and mortality, thus leading to poor outcomes in sepsis and surgical recovery [16]. Nutritional support and bowel rest helped prevent further gastrointestinal stress.

Definitive Surgical Intervention

Once stabilization has been achieved, the definitive treatment to eliminate the source of ongoing inflammation and prevent hemorrhage recurrence is elective cholecystectomy [17]. However, individual patient factors must be considered. The CHOCOLATE trial, which compared laparoscopic cholecystectomy versus percutaneous cholecystostomy in high-risk patients with acute cholecystitis, found no significant difference in mortality between the groups, although 45% of patients undergoing drainage eventually required delayed cholecystectomy due to clinical deterioration through an elective process. Surgery resulted in far fewer major complications, less frequent reinterventions, and lower rates of biliary disease, in addition to shorter hospital stays. Although the trial demonstrated that laparoscopic cholecystectomy was superior to percutaneous drainage, it also showed that percutaneous cholecystostomy followed by interval surgery is an acceptable and safe strategy for patients who are poor surgical candidates [18]. Therefore, the patient’s scheduled outpatient follow-up with hepatobiliary surgery is consistent with current best practices.

Prevention and risk reduction

Given the patient’s comorbidities, prevention revolves around minimizing iatrogenic injury and controlling infection. Several strategies may reduce the risk of similar complications in future cases. This includes imaging-guided cholecystostomy drainage using ultrasound or CT guidance, which can aid in avoiding vascular structures [10]. Strategic ERCP technique, with appropriate use of electrocautery during sphincterotomy and gentle instrumentation, is necessary to prevent vascular injury [9]. Close monitoring of stent position, with timely retrieval or exchange, helps prevent migration and supports peri-procedural optimization [12]. Finally, enhancing protocols for monitoring high-risk patients with diabetes, immunosuppression, or anticoagulation may facilitate earlier recognition of complications [13,16].

## Conclusions

This case illustrates hemobilia secondary to cystic artery hemorrhage following percutaneous cholecystostomy, a rare but serious complication in patients undergoing minimally invasive gallbladder decompression. The subtle and atypical presentation in this case, namely the absence of classic gastrointestinal bleeding, highlights the importance of clinical vigilance in post-procedural patients. Hemobilia should be considered in patients with new-onset bloody cholecystostomy output, unexplained anemia, or recurrent right upper quadrant symptoms. A multidisciplinary approach involving gastroenterology, interventional radiology, surgery, and critical care is essential. CT angiography remains the gold standard for diagnosis, while transcatheter arterial embolization or angiogram with stent placement provides a minimally invasive and highly effective treatment modality. Ultimately, elective cholecystectomy is essential to remove the underlying inflammatory source and prevent recurrence. Increasing awareness of this complication is crucial, especially as percutaneous approaches become more common in high-risk surgical patients. Early recognition and prompt intervention can significantly improve outcomes and reduce the risk of life-threatening hemorrhage.
